# Assessment of Autozygosity Derived From Runs of Homozygosity in Jinhua Pigs Disclosed by Sequencing Data

**DOI:** 10.3389/fgene.2019.00274

**Published:** 2019-03-28

**Authors:** Zhong Xu, Hao Sun, Zhe Zhang, Qingbo Zhao, Babatunde Shittu Olasege, Qiumeng Li, Yang Yue, Peipei Ma, Xiangzhe Zhang, Qishan Wang, Yuchun Pan

**Affiliations:** ^1^Department of Animal Science, School of Agriculture and Biology, Shanghai Jiao Tong University, Shanghai, China; ^2^Shanghai Key Laboratory of Veterinary Biotechnology, Shanghai, China

**Keywords:** pig, runs of homozygosity, inbreeding coefficients, selection, animal breeding

## Abstract

Jinhua pig, a well-known Chinese indigenous breed, has evolved as a pig breed with excellent meat quality, greater disease resistance, and higher prolificacy. The reduction in the number of Jinhua pigs over the past years has raised concerns about inbreeding. Runs of homozygosity (ROH) along the genome have been applied to quantify individual autozygosity to improve the understanding of inbreeding depression and identify genes associated with traits of interest. Here, we investigated the occurrence and distribution of ROH using next-generation sequencing data to characterize autozygosity in 202 Jinhua pigs, as well as to identify the genomic regions with high ROH frequencies within individuals. The average inbreeding coefficient, based on ROH longer than 1 Mb, was 0.168 ± 0.052. In total, 18,690 ROH were identified in all individuals, among which shorter segments (1–5 Mb) predominated. Individual ROH autosome coverage ranged from 5.32 to 29.14% in the Jinhua population. On average, approximately 16.8% of the whole genome was covered by ROH segments, with the lowest coverage on SSC11 and the highest coverage on SSC17. A total of 824 SNPs (about 0.5%) and 11 ROH island regions were identified (occurring in over 45% of the samples). Genes associated with reproduction (*HOXA3, HOXA7, HOXA10*, and *HOXA11*), meat quality (*MYOD1, LPIN3*, and *CTNNBL1*), appetite (*NUCB2*) and disease resistance traits (*MUC4, MUC13, MUC20, LMLN, ITGB5, HEG1, SLC12A8*, and *MYLK*) were identified in ROH islands. Moreover, several quantitative trait loci for ham weight and ham fat thickness were detected. Genes in ROH islands suggested, at least partially, a selection for economic traits and environmental adaptation, and should be subject of future investigation. These findings contribute to the understanding of the effects of environmental and artificial selection in shaping the distribution of functional variants in the pig genome.

## Introduction

Autozygosity refers to homozygosity in which the two alleles are identical by descent (IBD). It can result from several different phenomena, such as genetic drift, consanguineous matings, population bottleneck, as well as natural and artificial selection (Curik et al., 2014). Jinhua pig, as a valuable natural resource, is a well-known indigenous breed in eastern China that has evolved as a pig breed with excellent meat quality, greater disease resistance, higher prolificacy and greater adaptability to hot and humid climate (Gao et al., 2014). Due to their superior meat quality, Jinhua pigs have been used for the production of a famous ham brand called Jinhua Ham, which is a famous ham in China (Miao et al., 2009). The number of Jinhua pigs has been decreasing in the last two decades as a result of large import of Western pig breeds to improve leanness rate of pork (China National Commission of Animal Genetic Resources, 2011). A deficient control of inbreeding may lead to a reduction of the genetic variability and therefore of the effective population size (N_e_), a key parameter that influences the conservation planning and determines the rate of change in the composition of a population caused by genetic drift (Charlesworth, 2009). In addition, inbreeding may also increase the frequency of autozygosity for deleterious alleles with the consequent reduction in individual performance (Ouborg et al., 2010). For these reasons, there is a growing interest in characterizing and understanding inbreeding and autozygosity in Jinhua pigs. This would help to better preserve the genetic diversity and allow long-term viability of breeding programs of this breed.

Runs of homozygosity (ROH) are contiguous homozygous segments of the genome where the two haplotypes inherited from the parents are identical (Gibson et al., 2006). The development of high-density single nucleotide polymorphism (SNP) markers to scan the genome for ROH has been proposed as a proxy for the detection of genomic regions where a reduction in heterozygosity has occurred (Howrigan et al., 2011). Nowadays, whole genome inbreeding estimated from ROH is considered as a powerful method to distinguish between recent and ancient inbreeding (Keller et al., 2011). As the expected length of a ROH is equal to 1/2*G* Morgan, where *G* is the number of generations since the common ancestor (Thompson, 2013), the number of generations can be inferred from the length and frequency of ROH (Howrigan et al., 2011). The autozygosity, based on ROH, can help to improve the understanding of inbreeding depression of a trait (Keller et al., 2011) and also help to identify genes associated with traits of economic interest present in these ROH island regions (Purfield et al., 2017). In addition, given the stochastic nature of recombination, the occurrence of ROH is not randomly distributed across the genome, and ROH islands across a large number of samples may be the result of selective pressure (Zavarez et al., 2015). Recently, ROH has been used to explore signatures of selection in cattle (Peripolli et al., 2018), chicken (Marchesi et al., 2018), and sheep (Mastrangelo et al., 2017), but less commonly in pig, especially Chinese indigenous pigs such as the Jinhua pigs.

In this study, we investigated the occurrence and distribution of ROH in a sample of 202 Jinhua pigs, in order to characterize genome-wide autozygosity levels and to detect potential ROH islands that may provide insights into past events of selection in this population. In addition, other parameters to address the levels of genetic variability, including N_e_ and different measures of inbreeding from pedigree and genomic information, were also investigated. For that, we used genotyping by genome reducing and sequencing (GGRS) (Chen et al., 2013), which was successfully applied to evaluate genetic diversity in Chinese indigenous pig breeds in the Taihu region (Wang et al., 2015).

## Materials and Methods

### Population and Sequencing

Ear tissue samples were collected from 202 Jinhua pigs (189 females and 13 males) from conservation pig farms in Zhejiang province. Those pigs were born between 2014 and 2017, with an average depth of about four generations. A commercial kit (Lifefeng Biotech, Co., Ltd., Shanghai, China) was used to extract genomic DNA, and verified the integrity and purity of DNA by agarose gel electrophoresis and the A260/280 ratio. The Genomic DNA samples were genotyped using the GGRS protocol (Chen et al., 2013). Quality control (QC) of ∼1.4 billion raw reads were performed using NGS QC Toolkit v2.3 (Patel and Jain, 2012). In this study, we mapped the clean sequencing reads to the latest released pig reference genome (Sscrofa11.1) using BWA (Li and Durbin, 2010). SNP calling was performed using SAMTOOLS v0.1.19 and the missing genotypes were imputed using BEAGLE (Howie et al., 2009; Li et al., 2009). Additional quality controls were applied following Xiao et al. (2017). These included a minimum number of samples genotyped > 30%, a calling quality > 20 (99% accuracy), and a minor allele frequency (MAF) ≥ 5%. SNPs mapped to sex chromosomes were excluded from the analyses.

To determine novel variants in our sequence data, we compared the identified SNPs with the dbSNP data (Build 152^1^). These SNPs were annotated according to the Ensembl pig gene annotation set (Ensembl release 92^2^) as previously reported by Wang et al. (2015).

### Genetic Diversity

Observed (H_o_) and expected heterozygosity (H_e_) were estimated using PLINK v1.07 (Purcell et al., 2007). The N_e_ was estimated using SNeP v1.1 (Barbato et al., 2015). This approach simultaneously estimated historical effective population size based on the relationship between LD, N_e_, and recombination rate:

(1) $$N_{e(t)}={\left(4f(c_{t})\right)}^{-1}(E[r_{adj}^{2}|c_{t}{|}^{-1}-\alpha )$$

where *N*_e(t)_ is the effective population size t generations ago, calculated as *t* = (2*f* (*c_t_*))^-1^; c_t_ is the recombination rate for a specific physical distance between SNPs, which was estimated using Sved and Feldman (1973); *f* is the Haldane mapping function built between recombination rate and genetic distance measured by Morgan; $r_{\mathrm{adj}}^{2}$ is the LD value corrected for sample size and *α* is a correction for the occurrence of mutations.

Linkage disequilibrium between SNP pairs was estimated using PLINK v1.07 (Purcell et al., 2007). Haplotype blocks were obtained with a confidence intervals algorithm and with the software Haploview (Barrett, 2009), which was also used to visualize haplotype patterns.

### Measure of Runs of Homozygosity

Runs of homozygosity were identified for each individual using PLINK v1.07 (Purcell et al., 2007). The default parameter –*homozyg* were used to define ROH (Peripolli et al., 2018) and the following criteria were chosen: (1) a sliding window of 50 SNPs across the genome; (2) one heterozygous and five missing calls were allowed per window to account for genotyping error; (3) the minimum number of consecutive SNPs included in a ROH was set to 100; (4) to exclude short ROH that was derived from strong LD, the minimum length for a ROH was set to 1 Mb (Purfield et al., 2012); (5) the required minimum SNP density to define a ROH was 1 SNP per 50 kb. Considering an approximate genetic distance of 1 cM each 1 Mb (Zanella et al., 2016), a minimum ROH length of 1 Mb was expected to capture inbreeding up to 50 ancestral generations.

### Pedigree and Genomic Inbreeding Coefficients

Different types of inbreeding coefficients were estimated based on pedigree and genomic information. Pedigree-based inbreeding coefficients (F_PED_) for all pigs were estimated using R package “*pedigree.*”

Genomic inbreeding for each animal was estimated from ROH (F_ROH_), as the ratio of the total length of genome covered by ROH to the total length of the genome covered by SNPs or sequences, as proposed by McQuillan et al. (2008):

(2) $$F_{\mathrm{ROH}}=\frac{L_{ROH}}{L_{auto}},$$

in which L_ROH_ is the total length of an individual’s ROH and L_auto_ is the length of the autosomal genome covered by the SNPs, which was 2.26 Gb in our study. For each animal, four ROH estimates were calculated based on lengths from sequence data as the proportion of its genome: ROH > 10 Mb (F_ROH__>__10__Mb_), 5–10 Mb (F_ROH5-10_ _Mb_), 1–5 Mb (F_ROH1-5_ _Mb_), and ROH > 1 Mb (F_ROH_all_), corresponding to 5 generations, 5 to 10 generations, 10 to 50 generations, and 50 generations, respectively.

In addition, three SNP-based estimates of inbreeding coefficients were calculated using the option –ibc from the GCTA software (Yang et al., 2011): the first estimator, F_SNP1_, was based on the variance of the additive genotypes (VanRaden, 2008); the F_SNP2_ estimate was calculated based on the homozygous excess; the third estimator, F_SNP3_, was calculated based on the correlation between uniting gametes (Wright, 1922). The formulae are as follows:

(3) $$F_{\mathrm{SNP1}}=\frac{1}{n}\sum_{i=1}^{n}\frac{{\left(Y_{i}-2p_{i}\right)}^{2}}{h_{i}}-1,$$

(4) $$F_{\mathrm{SNP2}}=1-\frac{1}{n}\sum_{i=1}^{n}\frac{Y_{i}(2-Y_{i})}{h_{i}},$$

(5) $$F_{\mathrm{SNP3}}=\frac{1}{n}\sum_{i=1}^{n}\frac{Y_{I}^{2}-Y_{i}(1+2p_{i})+2p_{i}^{2}}{h_{i}}$$

Where Y_i_ is the number of the reference allele copies for the i-th SNP, p_i_ is the frequency of this allele in the sample and h_i_ = 2p_i_ (1-p_i_), and n is the total number of SNPs. Note that these coefficients were corrected by the allele frequencies of the current population and they can take negative values (Yang et al., 2013), while F_PED_ and F_ROH_ ranged from 0 to 1. The inbreeding coefficients obtained by the eight methods were compared using Pearson’s correlation.

### Detection of Common Runs of Homozygosity

Genomic regions with reduced genetic diversity can be found in ROH islands, and high homozygosity around the ROH islands that might harbor targets of positive selection and are under strong selective pressure (Pemberton et al., 2012). To identify the genomic overlapping ROH regions, we calculated the proportion of the occurrences of a SNP in ROH by counting the number of times the SNP was detected in those ROH across individuals, and this was plotted against the position of the SNP along the chromosome. The genomic regions most commonly associated with ROH were identified by selecting the top 0.5% of the SNPs most commonly observed in ROH (Pemberton et al., 2012). A series of adjacent SNPs, merged to constitute ROH islands and genes within each ROH island, were further extracted using the BIOMART package (Durinck et al., 2005). To further analyze the functions of identified genes, Kyoto Encyclopedia of Genes and Genomes (KEGG) pathway and Gene Ontology (GO) enrichment analyses were performed using DAVID 6.8^3^. Only terms with a *p*-value less than 0.05 were considered as significant and listed.

### Detection of Selection Signatures Within Jinhua Pigs

To compare the selection signatures obtained from ROH, the integrated haplotype score (iHS) test was performed within Jinhua pigs. The iHS is a measure of the amount of extended haplotype homozygosity at a given SNP, designed to use phased genotypes to identify putative regions of recent or ongoing positive selection in genomes (Voight et al., 2006). The haplotype was phased using fastPHASE with default parameters (Scheet and Stephens, 2006). The derived haplotypes were then analyzed using the *rehh* v2.0 R package (Gautier et al., 2017) as previously reported by Bertolini et al. (2018). The iHS score was computed for each autosomal SNP, and values obtained were standardized so that they followed a standard normal distribution. To calculate the *p*-value at the genomic level, the scores for each SNP were transformed as p_iHS_ = - log_10_ [1 - 2|Φ (iHS) - 0.5|], where Φ(x) represents the Gaussian cumulative distribution function and p_iHS_ is the two sided *p*-value associated with the neutral hypothesis of no selection (Gautier et al., 2017). Corresponding to the threshold of 0.5% for ROH islands, the | iHS| scores higher than 2.81 (*p* < 0.005) were considered as putative signatures of selection (Cardoso et al., 2018). In this study, significant iHS signals were reported only for ROH islands.

## Results and Discussion

**FIGURE 1:**
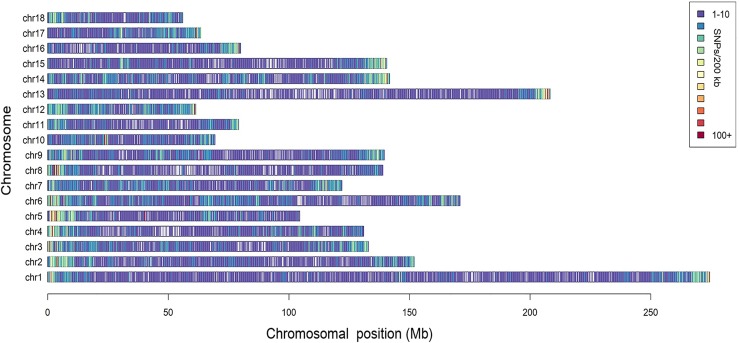
Distribution of the SNPs across the chromosomes. The x-axis denotes the chromosomal position (Mb), and the y-axis represents the chromosomes. The number of the SNPs present in each 200 kb genome block is expressed via colors.

A total of 166,661 informative SNPs satisfying the quality filters were obtained, 26,458 of which were identified as unreported in the pig SNP database of NCBI. The SNP density was about 1 SNP per 13.6 kb and they were equally distributed on each chromosome, with the exception of some isolated regions (Figure 1). According to the Ensembl pig gene annotation set (Ensembl release 92), 81,753 SNPs were mapped to gene regions, of which 8,092 were mapped as exons, and 4,481 were mapped as UTRs.

### Genetic Diversity

The average observed (H_o_) and expected (H_e_) heterozygosities in this Jinhua pig population were 0.312 ± 0.070 and 0.429 ± 0.057, respectively. The value of H_e_ was slightly higher than that reported by Chen et al. (2018) for Jinhua pig breed in Zhejiang province. The earliest legend regarding the Jinhua pigs may be traced back to approximately 1600 years ago (China National Commission of Animal Genetic Resources, 2011). Considering a generation interval of 1.5 years, the 1000 generations correspond to Jinhua populations 1500 years ago, approximately. The N_e_ was estimated from five to 1000 generations ago in our study. The results show that N_e_ has decreased through time, at a faster rate at 1,000 to 970 generations ago (Supplementary Figure S1). This finding could be explained by the domestication bottleneck caused by human-driven artificial selection approximately 1500 years ago. The effective population size in the last five generations was about 88 and was about 3018 in the 1000th generation. This value was larger than that reported by Xiao et al. (2017) for pig breeds in the Taihu region of China (ranging from 47 to 71) using the same method, therefore, this may indicate higher genetic diversity in Jinhua pigs.

### Pedigree and Genomic Inbreeding Coefficients Estimate

The average inbreeding coefficients estimated using different approaches are shown in Table 1. Incomplete pedigree failed to capture the influence of relatedness among founders from the base population, thus, the levels of inbreeding based on pedigree were expected to be lower than levels of inbreeding based on ROH and SNP-by-SNP (Table 1). The average F_ROH_ based on larger segments (0.041–0.053) was closer to F_PED_ (0.01) than the average F_SNP_. This was to some extent expected, given that the pedigree depth (about four generations) is in agreement with larger ROH segments. In addition, these two coefficients vary in the same range (0–1), while SNP-by-SNP coefficients estimated here can take lower or larger values. These low average F_ROH_ values of inbreeding suggest that recent inbreeding was low. However, according to SNP-by-SNP based coefficients, which reflect deviations of the observed inbreeding from the expected values in the current population, recent inbreeding seems to be considerably higher (0.262) than that based on pedigree and ROH.

**Table 1:** Descriptive statistics of the inbreeding coefficient based on pedigree (F_PED_), ROH (F_ROH1-5_ _Mb_, F_ROH5-10_ _Mb_, F_ROH>__10__Mb_, F_ROH_all_) and SNP-by-SNP (F_SNP1_, F_SNP2_, F_SNP3_).

Inbreeding coefficient	Mean	Min	Max	*SD*	*N*
F_PED_	0.010	0.000	0.137	0.025	202
F_ROH1-5_ _Mb_	0.073	0.036	0.118	0.017	202
F_ROH5-10_ _Mb_	0.041	0.007	0.076	0.015	202
F_ROH__>__10__Mb_	0.053	0.000	0.157	0.031	202
F_ROH_all_	0.168	0.053	0.291	0.052	202
F_SNP1_	0.262	-0.006	0.654	0.142	202
F_SNP2_	0.262	-0.148	0.581	0.181	202
F_SNP3_	0.262	-0.025	0.578	0.149	202


*Min, minimum; Max, maximum; SD, standard deviation; N, the numbers of animals.*

In contrast, the correlations between F_PED_ with all the genomic coefficients was low (from -0.009 to 0.053, Table 2), which may be indicative of a lack of power of F_PED_ to determine relatedness among founders from the base population (Visscher et al., 2006). These low correlations may also be affected by a poor and incomplete pedigree recording, as the base population assumed for F_PED_, based on long ROH and F_SNP_ was within the range of the last four generations. Correlations between genomic coefficients, based in ROH and in SNP-by-SNP approaches were considerably higher (from 0.218 to 0.698), increasing between coefficients computed from the same source of information, as expected (i.e., ROH segments or SNP-by-SNP information), thus suggesting that genotype-based estimates provide greater accuracy on relatedness as supported by previous studies (Purfield et al., 2012; Zanella et al., 2016).

**Table 2:** Correlation coefficients (lower panel) between pedigree-based inbreeding coefficients (F_PED_), four inbreeding coefficients based on different ROH lengths (F_ROH1-5_ _Mb_, F_ROH5-10_ _Mb_, F_ROH__>__10__Mb_, and F_ROH_all_) and three inbreeding coefficients based on SNP-by-SNP (F_SNP1_, F_SNP2,_ and F_SNP3_).

Correlation	F_PED_	F_ROH1-5Mb_	F_ROH5-10_ _Mb_	F_ROH__>__10Mb_	F_ROH_all_	F_SNP1_	F_SNP2_	F_SNP3_
F_PED_	1							
F_ROH1-5Mb_	0.049	1						
F_ROH5-10_ _Mb_	0.040	0.510^∗∗^	1					
F_ROH__>__10__Mb_	0.042	0.450^∗∗^	0.453^∗∗^	1				
F_ROH_all_	0.053	0.752^∗∗^	0.737^∗∗^	0.885^∗∗^	1			
F_SNP1_	0.029	0.293^∗∗^	0.218^∗∗^	0.295^∗∗^	0.339^∗∗^	1		
F_SNP2_	-0.009	0.628^∗∗^	0.490^∗∗^	0.573^∗∗^	0.698^∗∗^	0.702^∗∗^	1	
F_SNP3_	0.008	0.521^∗∗^	0.401^∗∗^	0.488^∗∗^	0.585^∗∗^	0.902^∗∗^	0.941^∗∗^	1


*^∗∗^Significantly different *p* < 0.01.*

Among the four inbreeding coefficients based on different ROH lengths, F_ROH_all_ (F_ROH>__1__Mb_) had higher correlations with F_SNP1_, F_SNP2,_ and F_SNP3_. A similar trend was also reported by Purfield et al. (2017) while studying six commercial meat sheep breeds. Among the three inbreeding coefficients based on SNP-by-SNP, F_SNP2_ had higher correlations with F_ROH1-5_ _Mb_, F_ROH5-10_ _Mb_, F_ROH__>__10Mb_ and all F_ROH_all_. These results corroborate previous results observed in cattle (Zhang et al., 2015a; Mastrangelo et al., 2016; Purfield et al., 2017). Similarly, Zhang et al. (2015a) also found that F_SNP2_ based on excess of homozygosity correlated relatively highly with F_ROH_ detected from 50k and sequence data. This trend may be due to the fact that both F_ROH_ and F_SNP2_ directly reflect homozygosity on the genome (Brito et al., 2017). F_SNP2_ (to some extent) capture all of the homozygosity, whereas, the F_ROH_ uses only ROH. Furthermore, the moderate to high correlations between F_ROH_ and the three other estimates of genomic inbreeding (F_SNP1_, F_SNP2_, and F_SNP3_) suggested that the proportion of the genome in ROH can be used as an accurate estimate of individual inbreeding levels (Purfield et al., 2012; Peripolli et al., 2018).

#### Genomic Distribution of Runs of Homozygosity

**FIGURE 2:**
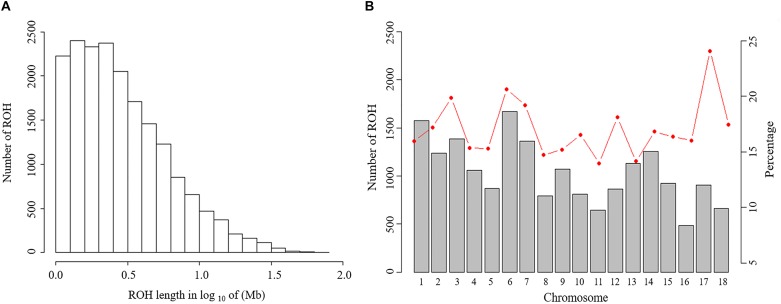
Distribution of the runs of homozygosity (ROH). **(A)** Distribution of ROH in different lengths (Mb). The values of length in Mb were transformed in log_10_. **(B)** Number of ROH longer than 1 Mb per chromosome (bars) and average percentage of each chromosome covered by ROH (red line).

The abundance and genomic distribution of ROH provide efficient information about the demographic history of livestock species (Bosse et al., 2012). In total, 18,690 ROH were identified in 202 individuals. The mean ROH length was 4.11 Mb and the longest segment, found in chromosome SSC1 had 72.45 Mb (2,237 SNPs). The distribution of ROH according to length is shown in Figure 2A. The descriptive statistics of ROH number and length by classes is given in Table 3. The total ROH number for Jinhua pigs was composed mostly of a high number of shorter segments (1–5 Mb), which accounted for approximately 77% of all ROH detected, and contributed about 43% of the cumulative ROH length. In contrast, larger ROH (>10 Mb), which were only 8% of all ROH, still covered about 32% of the total ROH length. These results revealed that both ancient (up to 50 generations ago) and recent (within the last five generations) inbreeding have had an impact on the genome of the Jinhua pig population.

**Table 3:** Descriptive statistics of runs of homozygosity (ROH) number and length (in Mb) by ROH length class (ROH 1-5 Mb, ROH 5-10 Mb, ROH > 10 Mb and total).

ROH length (Mb)	ROH number	Percent (%)	Mean length (Mb)	Standard deviation	Genome coverage (%)
1–5	14524	77.71	2.31	1.04	7.34
5–10	2753	14.73	6.85	1.38	4.12
>10	1413	7.56	17.24	7.79	5.33
Total (>1)	18690	100.00	4.11	4.73	16.80


For individuals, the relationship between total number of ROH and total length of the genome covered by ROH showed considerable variation among animals (Supplementary Figure S2). Individual ROH autosome coverage ranged from 5.32% (120.52 Mb) to 29.14% (659.87 Mb) in the Jinhua population. Similar distributions were also observed in other livestock species, such as sheep (Mastrangelo et al., 2017) and cattle (Peripolli et al., 2018).

For chromosomes, the number of ROH per chromosome and the percentage of chromosomes covered by ROH are shown in Figure 2B. The highest number of ROH per chromosome was on SSC6 (1,672 segments), whereas the lowest was on SSC16 (485 segments). On average, approximately 16.8% of the whole genome was under ROH segments, with the lowest coverage shown by SSC11 (14.0%) and the highest coverage of ROHs was on SSC17 (24.1%).

### Detection of Runs of Homozygosity Islands

Twenty seven percent of SNPs were comprised in ROH in at least 20% of individuals, thus suggesting that candidate autozygosity regions are present in this population. This finding was similar to that reported by Ferencakovic et al. (2013). The most frequent SNP detected in ROH (131 occurrences, 64.9%) mapped at ∼36 Mb in SSC3, according to the updated reference genome (Sscrofa11.1), although no genes have been currently mapped in this position, suggesting regulatory regions may be involved.

**FIGURE 3:**
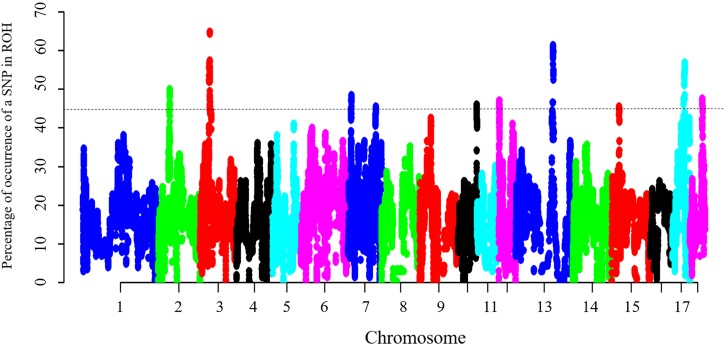
Manhattan plot of incidence of each SNP in the ROH across individuals. The dashed line represents the 45% threshold.

To identify the genomic regions that were most commonly associated with ROH in all individuals, the top 0.5% of SNPs with the highest occurrences (occurring in over 45% of the samples) in a ROH were considered as candidate SNPs (Figure 3). A total of 11 ROH island regions were identified, and the length of these regions ranged from 90 bp on SSC10 to 3.62 Mb on SSC3 (Table 4). The SNPs within these regions showed significantly higher linkage disequilibrium levels than the estimates obtained for the entire chromosome (Supplementary Table S1). On SSC8, we found the longest ROH cold-spot of 194 contiguous SNPs (12.56 Mb) that were not part of a ROH region in any of the individuals, thus suggesting a high heterozygosity region. This region might be produced by high recombination rates, or harboring loci with heterozygous advantage and under selection favoring high haplotype diversity. These results are in good agreement with low LD levels determined in these regions (Supplementary Figure S3) (Barrett, 2009). In the same way, ROH islands showed high levels of LD, as expected (Supplementary Figure S4).

### Candidate Genes Within Runs of Homozygosity Islands

Chromosome position, start and end position of ROH, ROH length, number of SNPs, and number of genes within the genomic regions of extended homozygosity are reported in Table 4. We found that some SNPs in ROH occurred in poor gene content regions. Some identified regions, such as that on SSC15, contained only one annotated gene, although it is longer than 1.3 Mb, either because the annotation of pig reference genome is still incomplete, or the genomic region is positioned in a non-coding region. A total number of 105 genes inside the ROH islands were analyzed using GO enrichment analysis. Supplementary Table S2 provides the chromosome position, start and end, gene name and Ensembl Gene ID for 105 genes. Supplementary Table S3 shows the significant GO terms and KEGG pathways; most of the genes were involved in metabolic pathways and biosynthetic process.

**Table 4:** List of genomic regions of extended homozygosity detected in Jinhua pigs.

CHR	Start (bp)	End (bp)	Length (bp)	Mean | iHS| value	SNPs	Genes
2	41304430	42207694	903264	2.56	94	15
3	33443006	37063749	3620743	2.01	174	9
7	11825920	12271785	445865	1.24	45	2
7	100881377	100912691	31314	2.94	16	1
10	67900330	67900420	90	0.00	3	0
12	2147273	2619710	472437	1.51	59	12
13	134231214	137556583	3325369	1.80	133	28
15	25936404	27246878	1310474	1.24	62	1
17	40820642	40961202	140560	1.00	6	1
17	43470563	44993583	1523020	1.71	141	7
18	45198816	46247949	1049133	1.66	91	29


*CHR, chromosome; SNPs, number of SNPs in each genomic region; Genes, number of genes in each genomic region.*

We also checked if the ROH islands overlapped with putative selection signatures in pigs in other literatures. We found that a ROH island at SSC7: 100881377–100912691 overlapped with gene *ADCK1*, involved in phosphate metabolism, which was in the selection signature region between Berkshire and Korean native pig breeds (Edea and Kim, 2014). A ROH island on SSC2 partially overlapped with a selection region for intramuscular fat and backfat thickness in two Duroc populations, which spanned three genes (*ABCC8, MYOD1*, and *PIK3C2A*) (Kim et al., 2015). The *MYOD1* gene in this region was also detected in the selection signatures between Jinhua pig group and European breeds group (Li et al., 2016).

In this paper, we focused on some of the most relevant genes within ROH that showed associations with several specific traits related to livestock breeding. Several candidate genes relating to reproduction traits were identified, such as the HOXA genes cluster: *HOXA3, HOXA7, HOXA10*, and *HOXA11* on SSC18, which affects embryo implantation and prolificacy traits (Bagot et al., 2000; Gao et al., 2010; Wu et al., 2013); *ROPN1*, involved in litter size trait in pigs (Lan et al., 2012); and *HNRNPA2B1*, which plays key roles in the preimplantation of pig embryo during elongation (Wilson et al., 2000). Some genes associated with specific traits related to meat quality were detected: *MYOD1*, which affects muscle fiber characteristics, the loin eye area and back fat thickness (Lee et al., 2012; Cepica et al., 2013); *LPIN3*, one member of lipin gene family associated with back-fat thickness in pigs (He et al., 2009), which are the important regulators in fat-tailed sheep with active lipid metabolism (Jiao et al., 2016); *CTNNBL1*, associated with porcine fat deposition and backfat traits (Yin et al., 2012). One gene was involved in appetite: *NUCB2*, which plays an important role in whole-body energy homeostasis and body weight at puberty by regulation of appetite (Lents et al., 2013). Most of genes we detected were involved in disease resistance traits: *MUC4, MUC13, MUC20, LMLN, ITGB5, HEG1, SLC12A8*, and *MYLK* on SSC13, were potential candidate genes for controlling the expression of the enterotoxigenic *Escherichia coli* (ETEC) with F4 fimbriae (F4ac) receptor (Huang et al., 2008; Jacobsen et al., 2010; Rampoldi et al., 2011; Fu et al., 2012; Ren et al., 2012). These genes played key roles in resistance to diarrhea by defending the attachment and adhesion of ETEC to porcine jejunal cells and in maintaining the epithelial barrier as well as immunity function (Zhou et al., 2013). Many studies have revealed that resistance to ETEC F4ac adhesion in pigs can be inherited as an autosomal recessive trait, and the pigs with homozygous genotype were usually resistant to ETEC F4ac (Rampoldi et al., 2011). The regional climate of the Jinhua pig is mainly subtropical with a weather condition that is hot and very humid, which is capable of inducing diarrhea especially during summer (Lyutskanov, 2011). Several researches have also shown that Jinhua pigs were more resistant to ETEC F4ac (Yan et al., 2009; Gao et al., 2014). These genes on SSC13 that display autozygosity in the Jinhua pigs may be linked to selection in response to hot and humid climate, as a result of local adaptation.

The Pig quantitative trait loci (QTL) database^4^ lists several QTL for reproduction, meat quality and immunity traits that overlapped these ROH islands (Quintanilla et al., 2011; Verardo et al., 2015; Zhang et al., 2016). In particular, some QTLs related to ham traits have already been reported: on SSC2, Cepica et al. (2013) identified significant QTL for ham weight (ID = 28220), Harmegnies et al. (2006) reported QTL for ham fat thickness (ID = 3938, ID = 3960, and ID = 3968); on SSC3, Choi et al. (2011) detected highly significant QTL for ham weight (ID = 21357), Stratil et al. (2006) reported QTL for ham meat weight (ID = 3102) and ham weight (ID = 3104).

In summary, the results show that the genomic regions that display autozygosity in the Jinhua pig breed are related to important production traits under selection, and possibly also help improve their adaptability to survive in hot and very humid environments.

### Selection Signature Analysis

**FIGURE 4:**
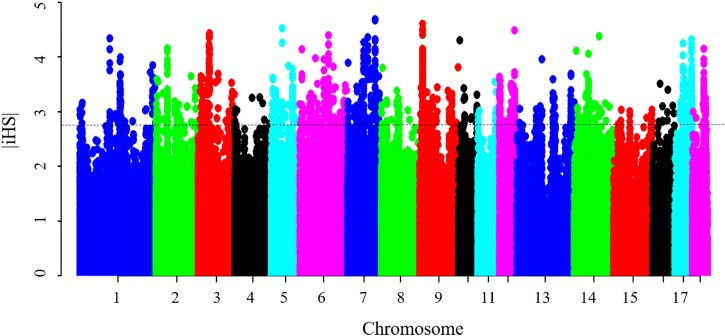
Genome-wide distribution of selection signatures detected by iHS. The dashed line represents the threshold levels of 0.5% (| iHS| = 2.81).

Results from the iHS test revealed that this coefficient had an average value of 0.77, with a maximum value of 4.69 on SSC7, thus indicating that the iHS values were not uniform across the genome. Figure 4 shows the genome-wide distribution of | iHS| values. The plots suggest evidence of selective forces in different regions of the genome. To compare these two methods, the occurrence of a SNP in a ROH was correlated with the SNP | iHS| value (Supplementary Figure S5). Significant moderate correlations were found between the iHS selection signature method and percentage of occurrence of a SNP in a ROH (Pearson’s correlation coefficient = 0.25, <0.0001). The average | iHS| values were also calculated in each ROH islands (Table 4). The result showed that mean | iHS| values of SNPs in each ROH islands (except one on SSC10) were higher than that across the genome (0.77) (Table 4).

There were 1,535 SNPs with *p*-value < 0.005 that harbored signatures of selection, 92 of which were found in ROH islands (Supplementary Table S4). A total of 42 candidate genes were found to overlap with these regions (Supplementary Table S5). It includes several genes mentioned above, such as *MYOD1, ADCK1, LPIN3, ITGB5, NUCB2, PIK3C2A*, and *ABCC8.* These genes obtained by the two methods should be given more consideration in further studies. The significant correlation between the iHS selection signature method and the percentage of occurrence of SNP in a ROH, in the present study and elsewhere (Zhang et al., 2015b), supports the hypothesis that the observed ROH islands are not only as a result of demography, but could also be due to selection.

## Conclusion

To our knowledge, this is the first study to describe the occurrence and distribution of ROH in the genome of Jinhua pigs. Autozygosity levels varied largely in this population, which has experienced both recent and historical inbreeding events. We have shown that, despite the low to moderate inbreeding levels in most animals, there were individuals with high inbreeding coefficients, indicating the need to account for inbreeding when planning mating strategy. Several genes within ROH islands are associated with adaptive and economic traits and should be the subject of future investigation. These findings may contribute to the understanding of the effects of environmental and artificial selection in shaping the distribution of functional variants in pig genome.

## Data Availability

All BAM data were deposited in the National Center for Biotechnology Information (NCBI) Sequence Read Archive (SRA). 202 samples are available under the Bioproject No. PRJNA525747.

## Ethics Statement

All experimental procedures were approved by the Institutional Animal Care and Use Committee of Shanghai Jiao Tong University, and all methods involved pigs were in accordance with the agreement of Institutional Animal Care and Use Committee of Shanghai Jiao Tong University (Contract No. 2011–0033).

## Author Contributions

YP, QW, and ZX designed the experiments. ZX, HS, QZ, XZ, QL, and YY performed the experiments. ZX, ZZ, and PM developed some of the analysis software. ZX wrote the manuscript with the help of BO. All authors read and approved the final manuscript.

## Conflict of Interest Statement

The authors declare that the research was conducted in the absence of any commercial or financial relationships that could be construed as a potential conflict of interest.

## Footnotes

1 ftp://ftp.ncbi.nlm.nih.gov/snp

2 ftp://ftp.ensembl.org/pub/release-92/gtf/sus_scrofa/

3 https://david.ncifcrf.gov/

4 https://www.animalgenome.org/cgi-bin/QTLdb/SS/index
