# Supplementary material for: Assessment of Autozygosity Derived From Runs of Homozygosity in Jinhua Pigs Disclosed by Sequencing Data
Source: Front Genet. 2019 Mar 28;10:274. doi: 10.3389/fgene.2019.00274 (PMC6448551; doi:10.3389/fgene.2019.00274)
Supplement: Supplementary file 1 [file Data_Sheet_1.docx]

Supplementary Material

**Assessment of autozygosity derived from runs of homozygosity in** **Jinhua pigs disclosed by sequencing data**

**Zhong Xu^1^, Hao Sun^1^, Zhe** **Zhang^1^, Qingbo Zhao^1^,** **Babatunde Shittu Olasege^1^, Qiumeng Li^1^, Yang Yue^1^, Peipei Ma^1^, Xiangzhe Zhang^1^, Qishan Wang^1*^, Yuchun Pan^1, 2*^**

^1^Department of Animal Science, School of Agriculture and Biology, Shanghai Jiao Tong University, Shanghai 200240, PR China

^2^Shanghai Key Laboratory of Veterinary Bio-technology, Shanghai 200240, PR China

***Correspondence:**

Yuchun Pan;

Qishan Wang

E-mail: [panyuchun1963@aliyun.com](mailto:panyuchun1963@aliyun.com)

[wangqishan@sjtu.edu.cn](mailto:wangqishan@sjtu.edu.cn)

1. **Supplementary Figures**

**
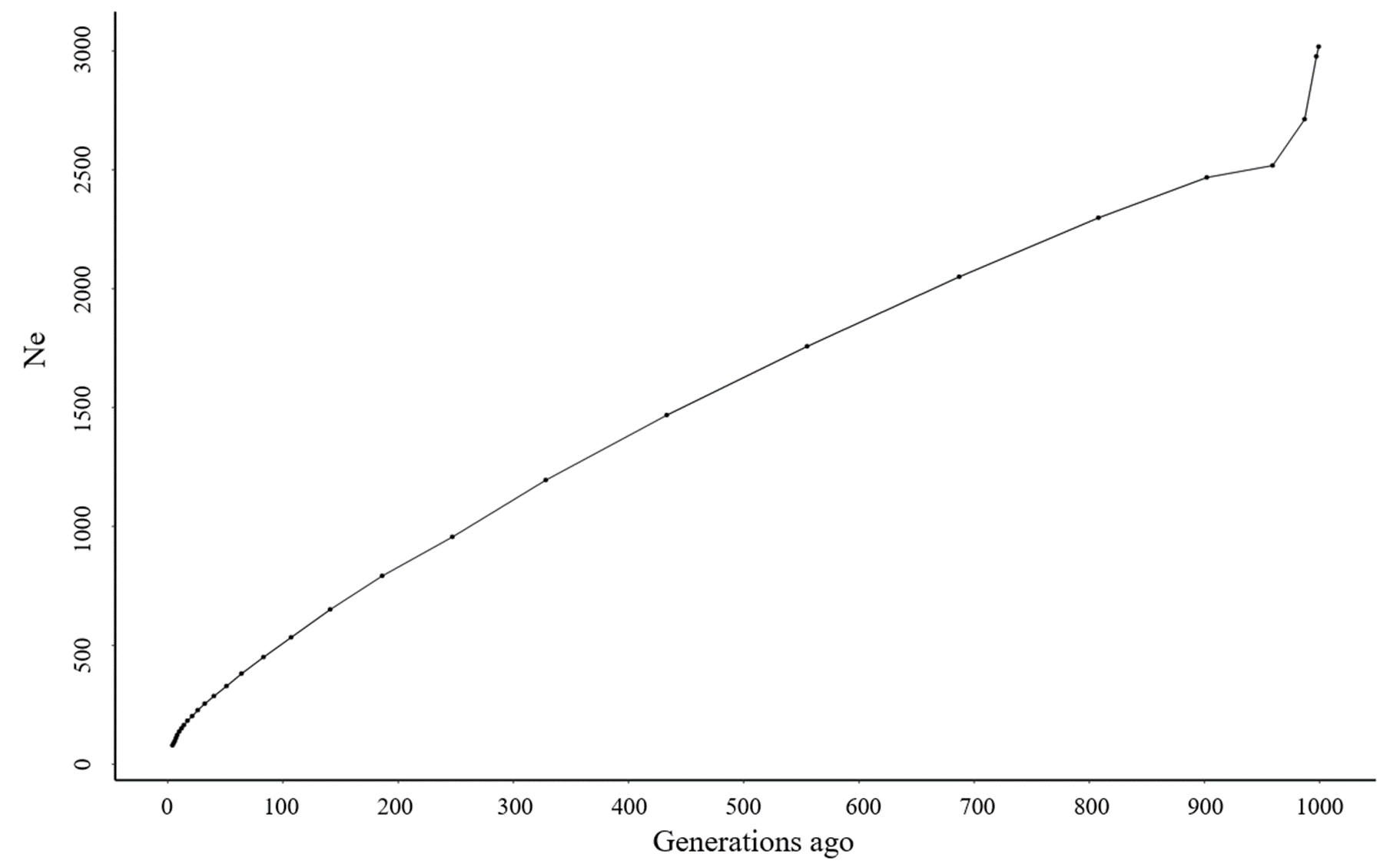
**

**Figure S1.** Estimated effective population size (N_e_) across generations for Jinhua pigs.


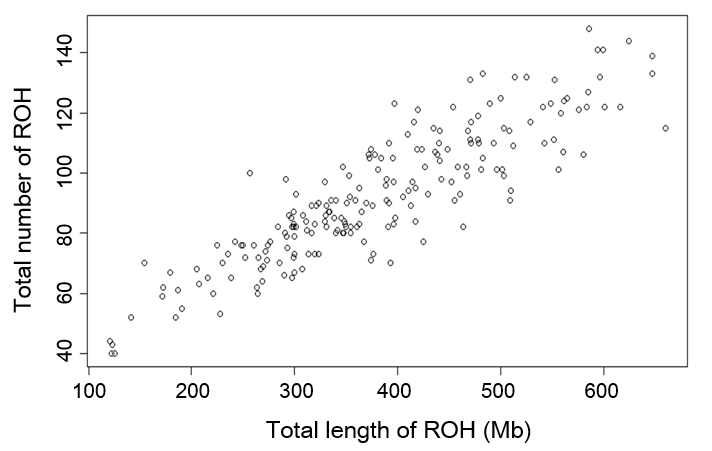


**Figure S2.** Total number of runs of homozygosity (ROH) longer than 1 Mb and total length of genome (Mb) covered by ROH segments per individual. Each dot represents an individual.


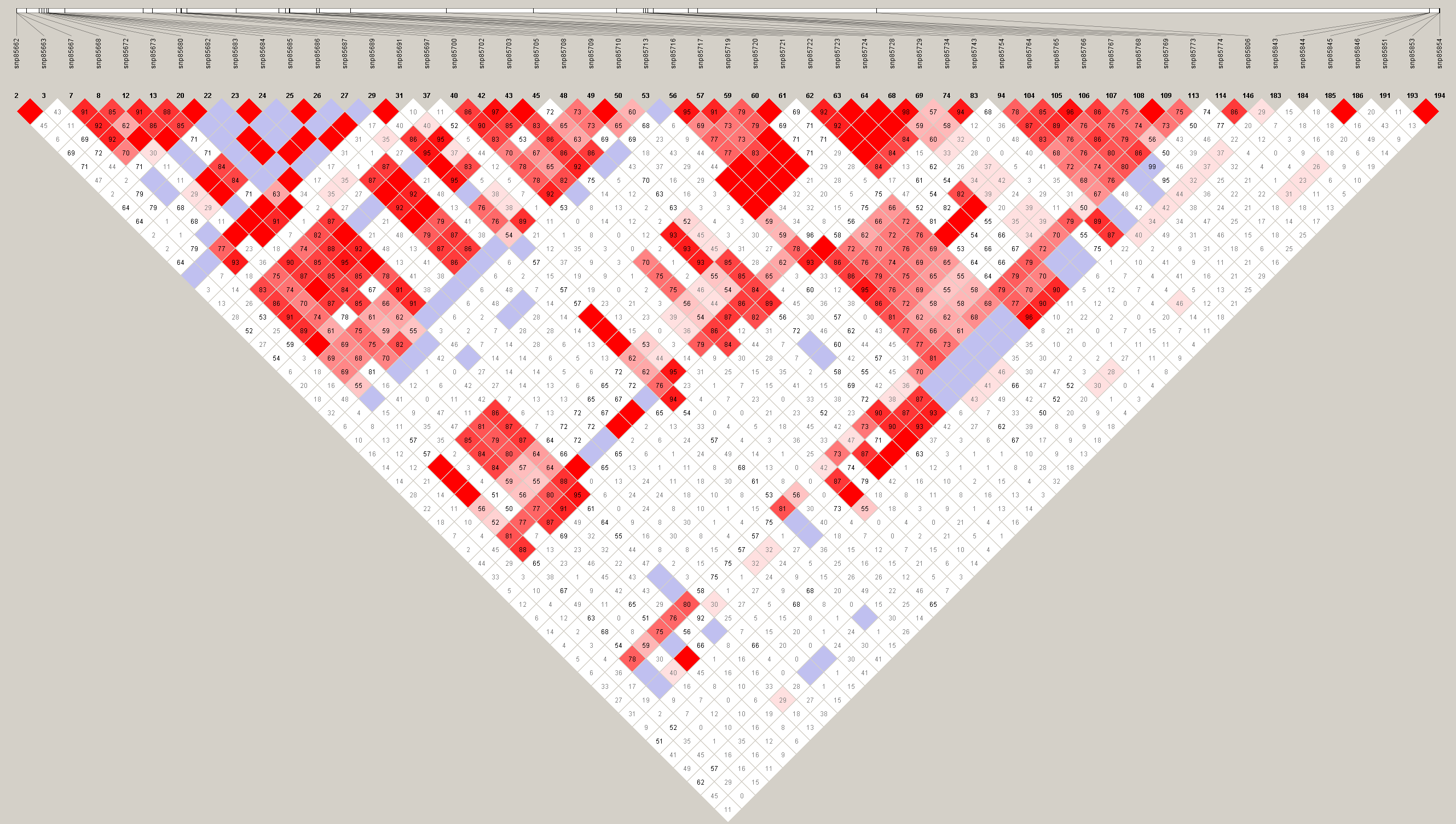


**Figure S3.** Linkage disequilibrium plot using Haploview of SNPs on chromosome 8 that did not appear in a ROH.

**
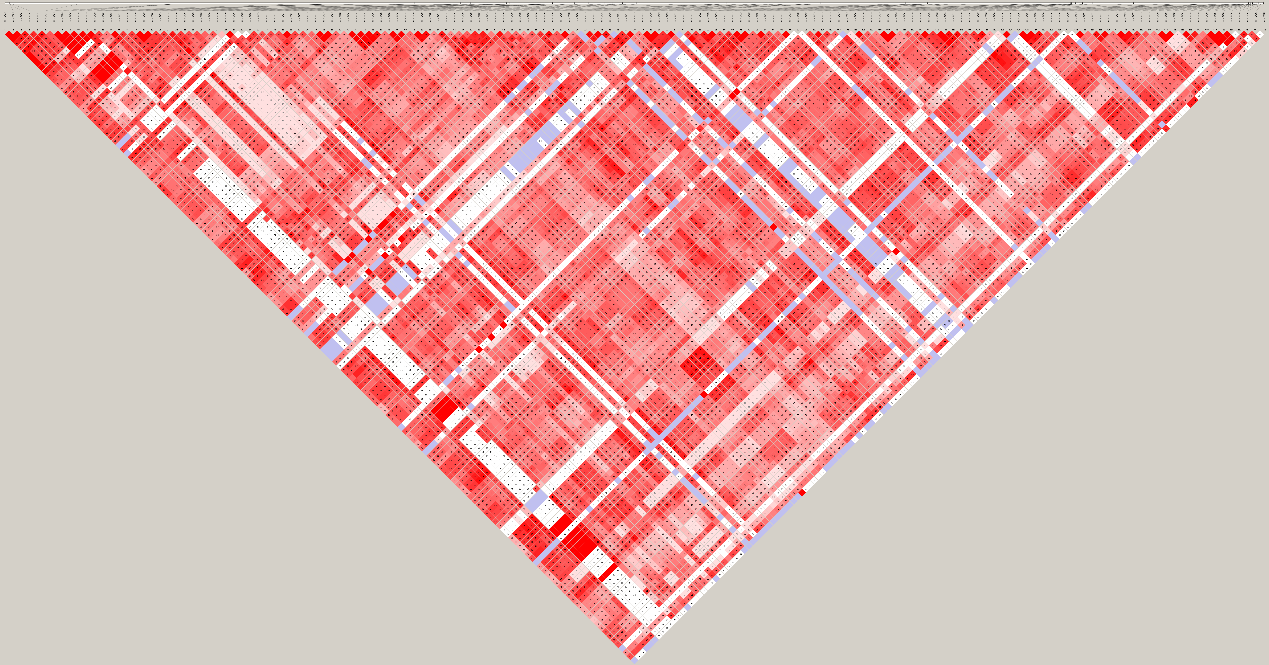
**

**Figure S4.** Linkage disequilibrium plot using Haploview of SNPs in ROH islands located on chromosome 13.


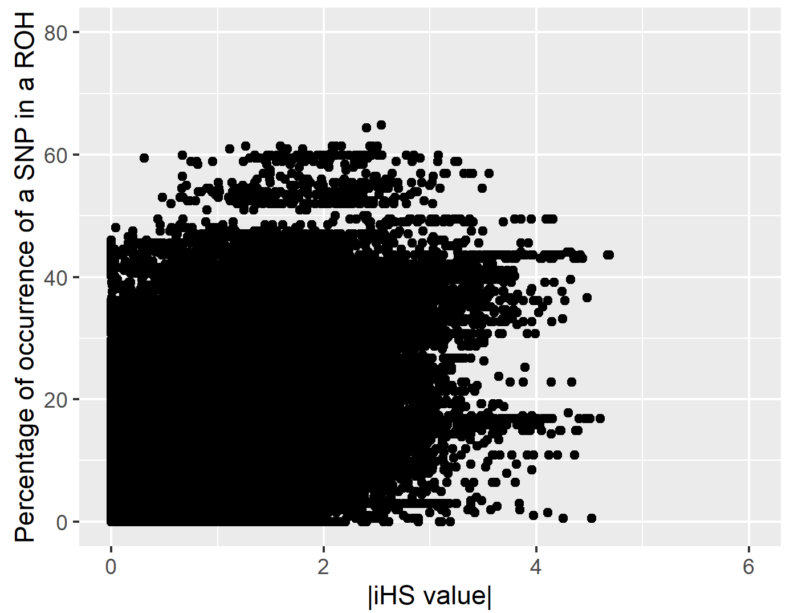


**Figure S5.** Correlation between |iHS| values versus the frequency of a SNP in a run of homozygosity. (Pearson’s correlation coefficient = 0.25; p < 0.001).

1. **Supplementary Tables**

**Table S1.** Mean linkage disequilibrium values for ROH islands and entire chromosome. Mean linkage disequilibrium (LD) values for each autosome and within each ROH island were estimated considering a physical distance lower than 100 kb between SNPs. The statistical significance was calculated by the Mann-Whitney U test.

| Chr | LD (Autosome) | | Start(bp) | End(bp) | LD (ROH island) | P-value |
| --- | --- | --- | --- | --- | --- | --- |
| 2 | 0.168 | 41304430 | | 42207694 | 0.290 | <0.001 |
| 3 | 0.178 | 33443006 | | 37063749 | 0.330 | <0.001 |
| 7 | 0.170 | 11825920 | | 12271785 | 0.299 | <0.001 |
| 7 | 0.170 | 100881377 | | 100912691 | 0.514 | <0.001 |
| 10 | 0.162 | 67900330 | | 67900420 | 0.649 | <0.001 |
| 12 | 0.171 | 2147273 | | 2619710 | 0.301 | <0.001 |
| 13 | 0.175 | 134231214 | | 137556583 | 0.466 | <0.001 |
| 15 | 0.167 | 25936404 | | 27246878 | 0.245 | <0.001 |
| 17 | 0.183 | 40820642 | | 40961202 | 0.426 | <0.001 |
| 17 | 0.183 | 43470563 | | 44993583 | 0.286 | <0.001 |
| 18 | 0.180 | 45198816 | | 46247949 | 0.254 | <0.001 |

**Table S2.** List of 105 potential candidate genes under directional selection in the Jinhua pigs.

| Chr | Gene Start (bp) | Gene End (bp) | Gene Name | Ensembl Gene ID |
| --- | --- | --- | --- | --- |
| 2 | 41028615 | 41356463 | *SAAL1* | ENSSSCG00000035393 |
| 2 | 41359655 | 41407745 | *KCNC1* | ENSSSCG00000013374 |
| 2 | 41421795 | 41424482 | *MYOD1* | ENSSSCG00000013375 |
| 2 | 41498499 | 41589691 | *OTOG* | ENSSSCG00000013376 |
| 2 | 41525258 | 41525369 | *SNORD89* | ENSSSCG00000020482 |
| 2 | 41605032 | 41645713 | *USH1C* | ENSSSCG00000013377 |
| 2 | 41666909 | 41756703 | *ABCC8* | ENSSSCG00000013378 |
| 2 | 41782038 | 41830532 | *NUCB2* | ENSSSCG00000013380 |
| 2 | 41933429 | 42022209 | *PIK3C2A* | ENSSSCG00000025406 |
| 2 | 42031806 | 42031896 | *SNORD14* | ENSSSCG00000018117 |
| 2 | 42031806 | 42031896 | *SNORD14B* | ENSSSCG00000018117 |
| 2 | 42033023 | 42033113 | *SNORD14A* | ENSSSCG00000018524 |
| 2 | 42064271 | 42064433 | *U1* | ENSSSCG00000019870 |
| 2 | 42064271 | 42064433 | *RNU1-51P* | ENSSSCG00000019870 |
| 2 | 42081768 | 42312689 | *PLEKHA7* | ENSSSCG00000013382 |
| 3 | 33626975 | 33655272 | *C16orf72* | ENSSSCG00000028454 |
| 3 | 33753800 | 33810465 | *USP7* | ENSSSCG00000007907 |
| 3 | 33834397 | 33847942 | *CARHSP1* | ENSSSCG00000026710 |
| 3 | 33857062 | 33892469 | *PMM2* | ENSSSCG00000021236 |
| 3 | 33892550 | 33895698 | *TMEM186* | ENSSSCG00000033311 |
| 3 | 33908963 | 34017770 | *ABAT* | ENSSSCG00000007909 |
| 3 | 34035904 | 34055464 | *METTL22* | ENSSSCG00000007914 |
| 3 | 34083997 | 34095247 | *TMEM114* | ENSSSCG00000007915 |
| 3 | 34935770 | 35326206 | *RBFOX1* | ENSSSCG00000007917 |
| 7 | 12117240 | 12137926 | *MYLIP* | ENSSSCG00000001063 |
| 7 | 12254388 | 12294555 | *GMPR* | ENSSSCG00000001064 |
| 7 | 100777134 | 100902638 | *ADCK1* | ENSSSCG00000002408 |
| 12 | 2087484 | 2212102 | *RNF213* | ENSSSCG00000017146 |
| 12 | 2220689 | 2241784 | *SLC26A11* | ENSSSCG00000021040 |
| 12 | 2241841 | 2249846 | *SGSH* | ENSSSCG00000017152 |
| 12 | 2249131 | 2275012 | *CARD14* | ENSSSCG00000017154 |
| 12 | 2285579 | 2311745 | *EIF4A3* | ENSSSCG00000017155 |
| 12 | 2316493 | 2336988 | *GAA* | ENSSSCG00000017156 |
| 12 | 2341574 | 2384103 | *CCDC40* | ENSSSCG00000017158 |
| 12 | 2401816 | 2455818 | *TBC1D16* | ENSSSCG00000017159 |
| 12 | 2521027 | 2527243 | *CBX4* | ENSSSCG00000017160 |
| 12 | 2562335 | 2567624 | *CBX8* | ENSSSCG00000034146 |
| 12 | 2569782 | 2580318 | *CBX2* | ENSSSCG00000024261 |
| 12 | 2611940 | 2619704 | *ENPP7* | ENSSSCG00000017163 |
| 13 | 134185794 | 134281086 | *MUC20* | ENSSSCG00000011851 |
| 13 | 134192534 | 134248023 | *MUC4* | ENSSSCG00000011850 |
| 13 | 134299209 | 134363695 | *RUBCN* | ENSSSCG00000011853 |
| 13 | 134363714 | 134403062 | *FYTTD1* | ENSSSCG00000027139 |
| 13 | 134405169 | 134539326 | *LRCH3* | ENSSSCG00000011854 |
| 13 | 134492460 | 134492586 | *SNORA31* | ENSSSCG00000020163 |
| 13 | 134495552 | 134495852 | *C8orf59* | ENSSSCG00000038638 |
| 13 | 134540368 | 134588271 | *IQCG* | ENSSSCG00000011855 |
| 13 | 134596155 | 134653434 | *LMLN* | ENSSSCG00000011857 |
| 13 | 134600010 | 134600114 | *RNU6-1238P* | ENSSSCG00000020428 |
| 13 | 134718351 | 134807146 | *OSBPL11* | ENSSSCG00000026753 |
| 13 | 134824068 | 134898690 | *SNX4* | ENSSSCG00000036828 |
| 13 | 134969668 | 135091568 | *ZNF148* | ENSSSCG00000011858 |
| 13 | 135105799 | 135249121 | *SLC12A8* | ENSSSCG00000011860 |
| 13 | 135296562 | 135358672 | *HEG1* | ENSSSCG00000011859 |
| 13 | 135416253 | 135442691 | *MUC13* | ENSSSCG00000011862 |
| 13 | 135467337 | 135590352 | *ITGB5* | ENSSSCG00000037905 |
| 13 | 135611861 | 135650636 | *UMPS* | ENSSSCG00000011864 |
| 13 | 135659354 | 135784890 | *KALRN* | ENSSSCG00000030362 |
| 13 | 136395615 | 136428595 | *ROPN1* | ENSSSCG00000028717 |
| 13 | 136395615 | 136428595 | *ROPN1B* | ENSSSCG00000028717 |
| 13 | 136438962 | 136493792 | *CCDC14* | ENSSSCG00000025349 |
| 13 | 136619308 | 136809673 | *MYLK* | ENSSSCG00000011867 |
| 13 | 136834120 | 136951061 | *HACD2* | ENSSSCG00000034786 |
| 13 | 136973000 | 137133977 | *ADCY5* | ENSSSCG00000027952 |
| 13 | 137173825 | 137250704 | *SEC22A* | ENSSSCG00000024904 |
| 13 | 137300051 | 137398480 | *PDIA5* | ENSSSCG00000011870 |
| 13 | 137455333 | 137586958 | *SEMA5B* | ENSSSCG00000011872 |
| 15 | 26882562 | 27362814 | *CNTNAP5* | ENSSSCG00000015726 |
| 17 | 40774120 | 40948915 | *CTNNBL1* | ENSSSCG00000007337 |
| 17 | 43710290 | 43799696 | *TOP1* | ENSSSCG00000007355 |
| 17 | 43810032 | 43844551 | *PLCG1* | ENSSSCG00000007356 |
| 17 | 43848368 | 43984240 | *ZHX3* | ENSSSCG00000032566 |
| 17 | 43993710 | 44006188 | *LPIN3* | ENSSSCG00000007359 |
| 17 | 44008021 | 44014677 | *EMILIN3* | ENSSSCG00000007358 |
| 17 | 44050008 | 44267340 | *CHD6* | ENSSSCG00000007360 |
| 17 | 44718780 | 45135905 | *PTPRT* | ENSSSCG00000038990 |
| 18 | 45327711 | 45331169 | *EVX1* | ENSSSCG00000016696 |
| 18 | 45367175 | 45367340 | *HOTTIP_4* | ENSSSCG00000034325 |
| 18 | 45370826 | 45371173 | *HOTTIP_3* | ENSSSCG00000035220 |
| 18 | 45371257 | 45371628 | *HOTTIP_2* | ENSSSCG00000040574 |
| 18 | 45373440 | 45380300 | *HOXA13* | ENSSSCG00000029666 |
| 18 | 45384798 | 45384979 | *HOXA11-AS1_6* | ENSSSCG00000036209 |
| 18 | 45385759 | 45385967 | *HOXA11-AS1_5* | ENSSSCG00000031208 |
| 18 | 45386649 | 45386881 | *HOXA11-AS1_4* | ENSSSCG00000033449 |
| 18 | 45387455 | 45387551 | *HOXA11-AS1_3* | ENSSSCG00000034760 |
| 18 | 45387694 | 45387879 | *HOXA11-AS1_2* | ENSSSCG00000035144 |
| 18 | 45388176 | 45388271 | *HOXA11-AS1_1* | ENSSSCG00000031379 |
| 18 | 45388574 | 45392573 | *HOXA11* | ENSSSCG00000016698 |
| 18 | 45393945 | 45402617 | *HOXA10* | ENSSSCG00000021204 |
| 18 | 45408457 | 45411913 | *HOXA9* | ENSSSCG00000028997 |
| 18 | 45417132 | 45421017 | *HOXA7* | ENSSSCG00000016701 |
| 18 | 45421658 | 45432918 | *HOXA5* | ENSSSCG00000016703 |
| 18 | 45443407 | 45444948 | *HOXA4* | ENSSSCG00000016704 |
| 18 | 45454213 | 45469740 | *HOXA3* | ENSSSCG00000016705 |
| 18 | 45471061 | 45472875 | *HOXA2* | ENSSSCG00000016706 |
| 18 | 45473490 | 45473636 | *HOTAIRM1_5* | ENSSSCG00000034336 |
| 18 | 45473646 | 45473744 | *HOTAIRM1_4* | ENSSSCG00000034323 |
| 18 | 45473763 | 45473820 | *HOTAIRM1_3* | ENSSSCG00000035169 |
| 18 | 45474496 | 45474709 | *HOTAIRM1_2* | ENSSSCG00000038342 |
| 18 | 45477324 | 45477448 | *HOTAIRM1_1* | ENSSSCG00000033523 |
| 18 | 45477548 | 45480413 | *HOXA1* | ENSSSCG00000016707 |
| 18 | 45562381 | 45791588 | *SKAP2* | ENSSSCG00000016708 |
| 18 | 46061611 | 46143002 | *SNX10* | ENSSSCG00000037598 |
| 18 | 46203102 | 46216449 | *HNRNPA2B1* | ENSSSCG00000036350 |
| 18 | 46217517 | 46250307 | *NFE2L3* | ENSSSCG00000026894 |

**Table S3.** GO terms and KEGG pathways enriched (p < 0.05) based on ROH islands.

| ID | Term | P value | Count |
| --- | --- | --- | --- |
| GO:0044707 | single-multicellular organism process | 2.33E-04 | 42 |
| GO:0009653 | anatomical structure morphogenesis | 5.50E-04 | 24 |
| GO:0048856 | anatomical structure development | 0.004599 | 36 |
| GO:0044767 | single-organism developmental process | 0.004629 | 36 |
| GO:0003008 | system process | 0.005098 | 18 |
| GO:0043229 | intracellular organelle | 0.010286 | 65 |
| GO:0048646 | anatomical structure formation involved in morphogenesis | 0.010682 | 12 |
| GO:0044424 | intracellular part | 0.017715 | 71 |
| GO:0005622 | intracellular | 0.022489 | 72 |
| GO:0009058 | biosynthetic process | 0.027941 | 37 |
| GO:0003700 | transcription factor activity, sequence-specific DNA binding | 0.043351 | 11 |
| KEGG pathway | Metabolic pathways | 0.01154 | 9 |

**Table S4.** List of overlapping SNPs deteced by iHS (p-value< 0.005) and ROH method.

| Chr | Position | Percentage of SNP in ROH (%) | iHS_value | *P*_value |
| --- | --- | --- | --- | --- |
| 2 | 41339892 | 46.04 | 3.086 | 0.002029 |
| 2 | 41345311 | 46.04 | -3.015 | 0.002566 |
| 2 | 41345350 | 46.04 | -3.061 | 0.002206 |
| 2 | 41345443 | 46.04 | -3.128 | 0.001762 |
| 2 | 41345458 | 46.04 | -3.103 | 0.001916 |
| 2 | 41345477 | 46.04 | -3.133 | 0.001732 |
| 2 | 41356731 | 46.04 | -3.077 | 0.002089 |
| 2 | 41357008 | 46.53 | 3.032 | 0.00243 |
| 2 | 41532873 | 49.5 | 3.172 | 0.001516 |
| 2 | 41540450 | 49.5 | -2.819 | 0.004813 |
| 2 | 41570073 | 49.5 | 3.223 | 0.001267 |
| 2 | 41571754 | 49.5 | 3.039 | 0.00237 |
| 2 | 41575674 | 49.5 | 3.053 | 0.002265 |
| 2 | 41611592 | 49.5 | 2.892 | 0.003833 |
| 2 | 41611638 | 49.5 | -3.164 | 0.001558 |
| 2 | 41611696 | 49.5 | -3.024 | 0.002497 |
| 2 | 41612756 | 49.5 | 3.149 | 0.001641 |
| 2 | 41612793 | 49.5 | 3.009 | 0.002624 |
| 2 | 41612798 | 49.5 | -3.251 | 0.001149 |
| 2 | 41612829 | 49.5 | 3.220 | 0.00128 |
| 2 | 41614540 | 49.5 | 3.272 | 0.001067 |
| 2 | 41622057 | 49.5 | 3.372 | 0.000747 |
| 2 | 41622233 | 49.5 | 3.794 | 0.000148 |
| 2 | 41625130 | 49.5 | -4.092 | 4.28E-05 |
| 2 | 41625371 | 49.5 | -4.137 | 3.52E-05 |
| 2 | 41625445 | 49.5 | 3.951 | 7.8E-05 |
| 2 | 41625479 | 49.5 | 4.158 | 3.22E-05 |
| 2 | 41628685 | 49.5 | 3.858 | 0.000114 |
| 2 | 41633655 | 49.5 | 2.916 | 0.003544 |
| 2 | 41633778 | 49.5 | -3.164 | 0.001556 |
| 2 | 41646030 | 49.5 | 3.290 | 0.001003 |
| 2 | 41659736 | 49.5 | -2.812 | 0.00492 |
| 2 | 41666007 | 49.5 | -3.135 | 0.001721 |
| 2 | 41702153 | 49.01 | 3.318 | 0.000906 |
| 2 | 41711158 | 49.01 | -2.878 | 0.003999 |
| 2 | 41727563 | 49.01 | 3.689 | 0.000225 |
| 2 | 41736208 | 49.01 | 3.124 | 0.001785 |
| 2 | 41736241 | 49.01 | 3.187 | 0.001438 |
| 2 | 41743749 | 49.01 | 3.334 | 0.000856 |
| 2 | 41751734 | 49.01 | -3.404 | 0.000664 |
| 2 | 41754757 | 49.01 | 3.368 | 0.000757 |
| 2 | 41754768 | 49.01 | -3.055 | 0.002253 |
| 2 | 41756401 | 49.01 | 3.022 | 0.00251 |
| 2 | 41756555 | 49.01 | 2.886 | 0.003904 |
| 2 | 41756562 | 49.01 | 2.995 | 0.002741 |
| 2 | 41756596 | 49.01 | 2.995 | 0.002741 |
| 3 | 33925863 | 55.45 | 2.828 | 0.004687 |
| 3 | 33925916 | 55.45 | 2.925 | 0.003445 |
| 3 | 33925921 | 55.45 | 3.016 | 0.002559 |
| 3 | 34036101 | 56.93 | -3.131 | 0.001743 |
| 3 | 34057977 | 56.93 | -3.365 | 0.000765 |
| 3 | 34058039 | 56.93 | -3.400 | 0.000675 |
| 3 | 34070034 | 56.93 | 3.318 | 0.000908 |
| 3 | 34070182 | 56.93 | 3.318 | 0.000908 |
| 3 | 34102100 | 56.93 | 2.914 | 0.003567 |
| 3 | 34102110 | 56.93 | 3.153 | 0.001614 |
| 3 | 34108445 | 56.93 | 3.557 | 0.000375 |
| 3 | 34110020 | 56.44 | -3.030 | 0.002448 |
| 3 | 34352217 | 52.48 | -2.946 | 0.003215 |
| 3 | 35017745 | 51.98 | 3.024 | 0.002494 |
| 3 | 35701113 | 54.46 | 3.489 | 0.000486 |
| 3 | 36281646 | 47.52 | 2.932 | 0.00337 |
| 3 | 36326226 | 47.52 | 3.390 | 0.0007 |
| 3 | 36326538 | 47.52 | 3.494 | 0.000476 |
| 3 | 36479495 | 46.04 | 3.338 | 0.000844 |
| 3 | 36486495 | 45.54 | 3.314 | 0.000921 |
| 7 | 100881377 | 45.05 | -3.173 | 0.001507 |
| 7 | 100881463 | 45.05 | -3.148 | 0.001645 |
| 7 | 100881600 | 45.05 | -3.198 | 0.001385 |
| 7 | 100881629 | 45.54 | -3.310 | 0.000931 |
| 7 | 100881632 | 45.54 | -3.256 | 0.00113 |
| 7 | 100881642 | 45.54 | -3.285 | 0.001018 |
| 7 | 100881665 | 45.54 | 3.856 | 0.000115 |
| 7 | 100886879 | 45.54 | 3.925 | 8.67E-05 |
| 7 | 100890916 | 45.54 | -2.927 | 0.003419 |
| 7 | 100895431 | 45.54 | 3.036 | 0.0024 |
| 7 | 100898604 | 45.54 | 3.457 | 0.000547 |
| 13 | 135670479 | 58.91 | 3.260 | 0.001112 |
| 13 | 135670508 | 58.91 | 3.260 | 0.001112 |
| 13 | 135670605 | 58.91 | 3.232 | 0.001229 |
| 13 | 135675855 | 58.91 | 2.832 | 0.004631 |
| 13 | 135675865 | 59.41 | 2.909 | 0.003627 |
| 13 | 136153790 | 58.91 | 3.072 | 0.002125 |
| 13 | 137012405 | 59.9 | 3.079 | 0.002074 |
| 13 | 137060020 | 59.9 | 2.848 | 0.004394 |
| 13 | 137521005 | 54.46 | 3.094 | 0.001973 |
| 13 | 137555095 | 53.96 | 2.877 | 0.004011 |
| 15 | 25958786 | 45.54 | 2.829 | 0.004665 |
| 17 | 43607721 | 53.47 | 2.816 | 0.004867 |
| 17 | 43995692 | 53.96 | 2.852 | 0.00435 |
| 17 | 43995747 | 53.96 | 2.864 | 0.004179 |
| 17 | 44415382 | 54.95 | 2.997 | 0.002729 |

**Table S5.** List of 42 potential candidate genes deteced by iHS (p-value< 0.005) and ROH method.

| Chr | Gene Start (bp) | Gene End (bp) | Gene Name | Ensembl Gene ID |
| --- | --- | --- | --- | --- |
| 2 | 41028615 | 41356463 | SAAL1 | ENSSSCG00000035393 |
| 2 | 41359655 | 41407745 | KCNC1 | ENSSSCG00000013374 |
| 2 | 41421795 | 41424482 | MYOD1 | ENSSSCG00000013375 |
| 2 | 41498499 | 41589691 | OTOG | ENSSSCG00000013376 |
| 2 | 41525258 | 41525369 | SNORD89 | ENSSSCG00000020482 |
| 2 | 41605032 | 41645713 | USH1C | ENSSSCG00000013377 |
| 2 | 41666909 | 41756703 | ABCC8 | ENSSSCG00000013378 |
| 2 | 41782038 | 41830532 | NUCB2 | ENSSSCG00000013380 |
| 2 | 41933429 | 42022209 | PIK3C2A | ENSSSCG00000025406 |
| 3 | 33753800 | 33810465 | USP7 | ENSSSCG00000007907 |
| 3 | 33834397 | 33847942 | CARHSP1 | ENSSSCG00000026710 |
| 3 | 33857062 | 33892469 | PMM2 | ENSSSCG00000021236 |
| 3 | 33892550 | 33895698 | TMEM186 | ENSSSCG00000033311 |
| 3 | 33908963 | 34017770 | ABAT | ENSSSCG00000007909 |
| 3 | 34035904 | 34055464 | METTL22 | ENSSSCG00000007914 |
| 3 | 34083997 | 34095247 | TMEM114 | ENSSSCG00000007915 |
| 3 | 34935770 | 35326206 | RBFOX1 | ENSSSCG00000007917 |
| 7 | 100679783 | 100710386 | ALKBH1 | ENSSSCG00000025906 |
| 7 | 100710470 | 100718302 | SLIRP | ENSSSCG00000028167 |
| 7 | 100718048 | 100749378 | SNW1 | ENSSSCG00000022932 |
| 7 | 100777134 | 100902638 | ADCK1 | ENSSSCG00000002408 |
| 7 | 100786306 | 100786409 | RNU6-1103P | ENSSSCG00000037944 |
| 7 | 100786306 | 100786409 | RNU6-1104P | ENSSSCG00000037944 |
| 7 | 100934986 | 100935249 | MRPS21 | ENSSSCG00000002409 |
| 7 | 101010263 | 101011488 | CDK1 | ENSSSCG00000033094 |
| 13 | 135467337 | 135590352 | ITGB5 | ENSSSCG00000037905 |
| 13 | 135611861 | 135650636 | UMPS | ENSSSCG00000011864 |
| 13 | 135659354 | 135784890 | KALRN | ENSSSCG00000030362 |
| 13 | 136834120 | 136951061 | HACD2 | ENSSSCG00000034786 |
| 13 | 136973000 | 137133977 | ADCY5 | ENSSSCG00000027952 |
| 13 | 137173825 | 137250704 | SEC22A | ENSSSCG00000024904 |
| 13 | 137300051 | 137398480 | PDIA5 | ENSSSCG00000011870 |
| 13 | 137455333 | 137586958 | SEMA5B | ENSSSCG00000011872 |
| 13 | 137638077 | 137739045 | DIRC2 | ENSSSCG00000036766 |
| 13 | 137739144 | 137809274 | HSPBAP1 | ENSSSCG00000011873 |
| 17 | 43410388 | 43411359 | MAFB | ENSSSCG00000032251 |
| 17 | 43710290 | 43799696 | TOP1 | ENSSSCG00000007355 |
| 17 | 43810032 | 43844551 | PLCG1 | ENSSSCG00000007356 |
| 17 | 43848368 | 43984240 | ZHX3 | ENSSSCG00000032566 |
| 17 | 43993710 | 44006188 | LPIN3 | ENSSSCG00000007359 |
| 17 | 44008021 | 44014677 | EMILIN3 | ENSSSCG00000007358 |
| 17 | 44050008 | 44267340 | CHD6 | ENSSSCG00000007360 |
